# A novel MAC protocol for power line communication with integrated NFC for smart home applications

**DOI:** 10.1038/s41598-024-82636-9

**Published:** 2024-12-30

**Authors:** G. Haridoss, J. Arun Pandian, K. Sivaranjani, L. Thanga Mariappan

**Affiliations:** 1 Department of Electronics and Communication Engineering , M.A.M College of Engineering and Technology , Trichy, India; 2 School of Computer Science Engineering and Information Systems , Vellore Institute of Technology , Vellore, India; 3 Department of Electronics and Communication Engineering , SRM TRP Engineering College , Trichy, India

**Keywords:** Power-line communication, Medium Access Control Protocol, Smart cities, Data, Transmission Security, Near Field Communication, Electrical and electronic engineering, Computer science, Information technology

## Abstract

Our day-to-day lives have become comfortable and sophisticated with many recent technologies. Likewise, today’s world has been enhanced by new innovative technologies. Everyone is moving towards smart cities and smart homes. Power-Line Communication (PLC) is a breakthrough communication technology that supports cost-effectiveness with maximum speed and reliable communication performance, producing no interference complications for wireless signal transmission. It operates over existing power line cables, avoiding other transmission mediums by using low-cost PLC chipsets. Transmission between two cities is performed using power grid technology. However, with a PLC chipset, data transmission is managed locally within towns and inside homes. To avoid inconveniences such as noise, significant signal reduction, and time period fluctuations, a novel Medium Access Control (MAC) protocol is proposed to improve the stability of the PLC network and data transmission efficiency. Using a Modified Intra-cluster MAC algorithm based on time allocation to enhance the reliability of access nodes in the PLC network is a novel attempt. Moreover, this work ensures enhancements in data transmission speed and security. Finally, the security-enhanced PLC network is integrated with Near Field Communication (NFC) technology for intelligent home applications.

## Introduction

Nowadays, the usage of smart devices everywhere is most common for all real-time applications. An adequate information and communication system is responsible for intelligent electric devices^1^. Also, the power line communication has expected conduit properties and implementation practicality^2^. Therefore, the consideration of optical communication as a PLC technology is vital for the advancement of smart grid technology^3^. In addition to that, electricity cables are present all over the place in our contemporary life expectancy, and hence they are harnessed for power line transmission technology. Thereby reducing the daily maintenance and investment for the extra infrastructure^4^. Therefore, the concept of the PLC network reached the highest place in its enhancement where the channel properties and the methodological encounters are sound learned and managed. The characteristics embrace resistivity dissimilarities, occurrence and time reliant on communication features, unrestrained connection, discharged secretions, the sound of light switches, and time hooked on output^5^.

However, the power line channel cannot transmit various signals such as speech, images, and video^6^. Similarly, the low voltage power line channel consists of different problems such as station blast, occurrence discerning reduction, output assemble difficulty, and multi-path consequence. These effects are undesirable because they produce maximum bit fault proportion, little accomplishment percentage, bulky signal reduction, and petite communication detachment of power line communication, leading to the unreliability of the entire announcement network^7^. Hence, maintaining the consistency of the power line communication network has one of the primary challenges that block its wide applications. Therefore, we need to concentrate more on the MAC layer, which contains the corporal layer, Logical Link Control (LLC) layer, and the network layer.

The power line communication network in the intelligent house uses Near Field Technology for low-power applications. Near field communication is a system of brief-series (radio-frequency), minimum-electric signal transmission through the wireless medium for automated devices, which permits them to interconnect with others by modest pitiful or to fetch them at identical nearby remoteness^8,9^. This data transmission performance is entitled ‘tap-in’ or ‘to tap and go’. The NFC data transmission decorum frequently happens among either two active devices such as cellular phones and palmtops or even amongst an NFC device and a passive (or unpowered) ‘tag’.

The main objective of the proposed MAC (Medium Access Control) protocol for power-line communication in smart cities is to enhance the reliability and efficiency of data transmission within smart home applications. The protocol aims to improve data communication effectiveness and system robustness by utilizing a modified intra-cluster MAC algorithm based on time allocation. This approach is designed to reduce data packet interference and ensure secure and efficient communication among various devices connected through power-line communication (PLC) networks.

The Major Contributions of this research are as follows:

(i) In this work, a modified MAC protocol has been proposed and constructed on the period to advance the data communication and the firmness of the system.
(ii) The average transmission delay of the proposed algorithm is calculated for maximum network utilization under different network sizes. The simulation result is taken for 10 different network sizes and set the communication rate of 20 kb/s for the length of a packet is 25 bytes.
(iii) Modified intra-cluster MAC technique based on time allocation to improve access node reliability in the PLC system. The time period distribution mechanism can reduce the probability of data packet interference.
(iv) This research also proposed a model for studying network stability, which depicts the relationship between network utilization and network size.
(v) The main contribution of our work is to provide a high success-rate of wireless communication for smart home applications. For example, the medical data in the house can be successfully reached by the user staying outside their home.
(vi) The success rate of the wireless communication has been achieved by time period allocation (clustering) algorithm.
(vii) The clustering of the transmission message can be done using the MAC protocol. The modified Intra-clustering MAC protocol achieved significantly less transmission delay and a high success rate of transmission.
(viii) The simulation results show that for big data packets, the proposed protocol exhibits much less transmission delay compared with other protocols. For example: for 180 nodes, there are 190 data packets have been delivered.
(ix) Similarly, the success rate of the wireless communication for 3 nodes achieves 90%, for 5 nodes success rate is 85% and for 9 nodes success rate is 60% from the simulation result.

The research article is organized as follows: Session 2 explains the general architecture of the MAC protocol-based PLC network, and Session 3 delivers the Architecture of Wireless Networks & Intra cluster MAC protocol. Session 4 explains the architecture of the PLC network and session 5 infers the state-of-art works. The proposed work & the simulation results have been illustrated with a modified Intra-cluster MAC protocol in session 6. Finally, the conclusion is given in session 7.

## Power line communication using MAC protocol

This session discusses the implementation of power line communication using Multiple Access Control protocols. Because of this construction, the network will be reachable where the wireless router signal could not process the area. To implement PLC with MAC protocol, broadband adaptation is required. As a broadband adapter, TP-LINK TL-WPA8630P has been used to operate up to 1200 Mbps. The TP-LINK adapter depends on the HomePlug AV2 specification. The proposed idea of using a power line as a wireless network is considered a better replacement for a wireless network. As stated above, the Wi-Fi network will be employed in the house power line so that intelligent control for other home appliances will also be carried out.

### Overview of the PLC & MAC

Smart homes are assumed to be a new trend to yield valuable services for the customers to reduce cost or energy and create a comfortable life. The smart home has the capacity of automatic control over the home appliances by turning off the devices during the absence of the user. Thereby the smart home application curtails the unnecessary usage of power. The smart home has the feature of automatic operation, and hence our life has become more accessible. Wireless communication engineering is presently the leading adapted technology for innovative home applications in state-of-the-art methods. But the wireless technology has its issues while signal spreading and exposure limits over a greater area. So, the requirement for another replacement technology is desired. Nowadays, Power Line Communication is getting attention for constructing broadband networks which bring increased data transfer rates based on power grids^10–16^. Also, power line communication has salient features over wireless technology for smart home automation, such as widening the network area. Moreover, there was no problem when the wall worked with conductive material. Hence, incorporating wireless technology with power line communication brought more exciting advantages to the innovative home design.

Therefore, this work combined the properties of power line communication and MAC (wi-fi) protocol in this work. In the existing system, the researchers used power line communication with Wi-Fi or power line communication with ZigBee^17,18^. Existing there are many protocols for efficiency and energy conservating are presented. Initially, the MAC protocol, such as IEEE 802.11, was unsuitable for wireless network communication. Since they are required to access the channel medium often, it consumes more energy^19^. Moreover, the same nodes have to transmit control packets to prevent collisions. On the other hand, Time Division Multiple Access (TDMA) network topologies assume all the secondary nodes within their transmission range of the controller node. This methodology provides efficient energy consumption over multi-hop routing employed for data transfer in the network. Similarly, the contention-based MAC protocol uses a node-level fairness approach to decrease energy usage. However, the contention-based MAC protocol does not fit for collision avoidance. The modified content-based MAC protocol in^20^reduces a considerable amount of energy by harnessing the feature of sleep mode operation if there are no used nodes present. Suppose many nodes want to send data to a particular node, utilizing RTS/CTS mechanism content as the medium for a selected node to transmit data. This will consume maximum energy compared to TDMA and MAC protocols. Likewise, PAMAS^21^is another protocol that automatically turns off unused nodes. And this method saves about 70% of energy. However, this method could not eliminate the collision. In addition to that, it uses two separate channels for its transmission. Hence it requires much cost, size, and complexity of the network design^22–25^. To utilize the features of TDMA technology, the nodes in the network must be synchronized. But the synchronization messages will consume a considerable amount of energy^26^. Hence, there is a need to propose a modified technique for an efficient energy-conservating scheme.

The Concept of virtual time-slotted channel hopping used in the proposed protocol allowing the devices to transmit data without overlapping and splits the communication into discrete time slots. This method improves the network efficiency and reduces the collisions. It incorporates feedback systems to ensure successful data transmission and also arranged the devices into clusters for better reciprocity. In this method the devices can switch between different channels during their assigned slots, enhancing reliability by avoiding interference.

There are various types of models in the particular MAC protocol. For instance, conventional power line communication runs on the MAC protocol of the IEEE 802.11 standard^27–29^. If the purpose of the network is to provide hybrid network technology, then the power line communication used IEEE 1905.1^30,31^. This present work discusses two significant signal-propagating methodologies: power line communication and wireless technology^32–34^. And at the same time, this project engrossed in the network properties assembled through the TP-LINK electricity cable wi-fi connecter equipment^35^. Concerning this proposed work, the wi-fi module is assumed to be the access point linked through Ethernet to the PLC/wi-fi switches TL-PA8010P PLC, which propagates the signal via PLC to the PLC/wi-fi plugs TL-WPA8630P^36–38^. As the System-on-chip (SOC) module is integrated with both the technology of Wi-Fi and PLC, it requires two modems^39^. Moreover, the selected modem has various features of producing throughput over 1 Gbps, a high rate of feasibility with Home Plug AV2, and the opportunity to arrange MIMO as a replacement for the furthermost commonly used SISO^40–42^. This modified Intra Cluster algorithm enhances the reliability of access nodes allocating specific time slots for each device, reducing collisions. It powerfully adjusts these time slots based on real-time network conditions. The algorithm prioritizes traffic and ensures timely delivery of important messages. It includes error detection mechanisms for immediate retransmission within allocated slots, enhancing data integrity. Overall performance is improved and gives room to add other devices.

The MAC protocol for Power Line Communication (PLC) can effectively handle other devices within the home. It uses an organised method with adaptive time slots which avoid transmission collisions and maximize communication efficiency. Contention-Free and Contention Periods assure that sensitive data can be sent unimpeded, and CSMA/CA design mitigates any possible simultaneous sends. By using the dynamic channel hopping technique the reliability can be improved and prevent the devices from interference circumstances. The information can be prioritised and retransmitted by traffic priority and error detection mechanism. Finally, the clustered architecture leads this network with reduced traffic, improved reliability and ensures the error free communication.

The primary motive for selecting this type of modem is that it is very convenient for commercial obtainability. The recombining of both the technologies, such as wireless and power line communication, induces some disturbance in communication. This session discussed the solution to overcome the problem and issue.

### Related work

The PLC network consists of many nodes for proper communication. The data link layer is accountable for communicating data between two systems. Its primary operations are,

- Data Link Layer.
- Multiple Access Control.

***Data link layer***

The function of data link control is to authority for unfailing dissemination of data through conveyance medium by exploiting methods like edging, fault authority, and flow control.

***Multiple access control***

**Fig. 1:**
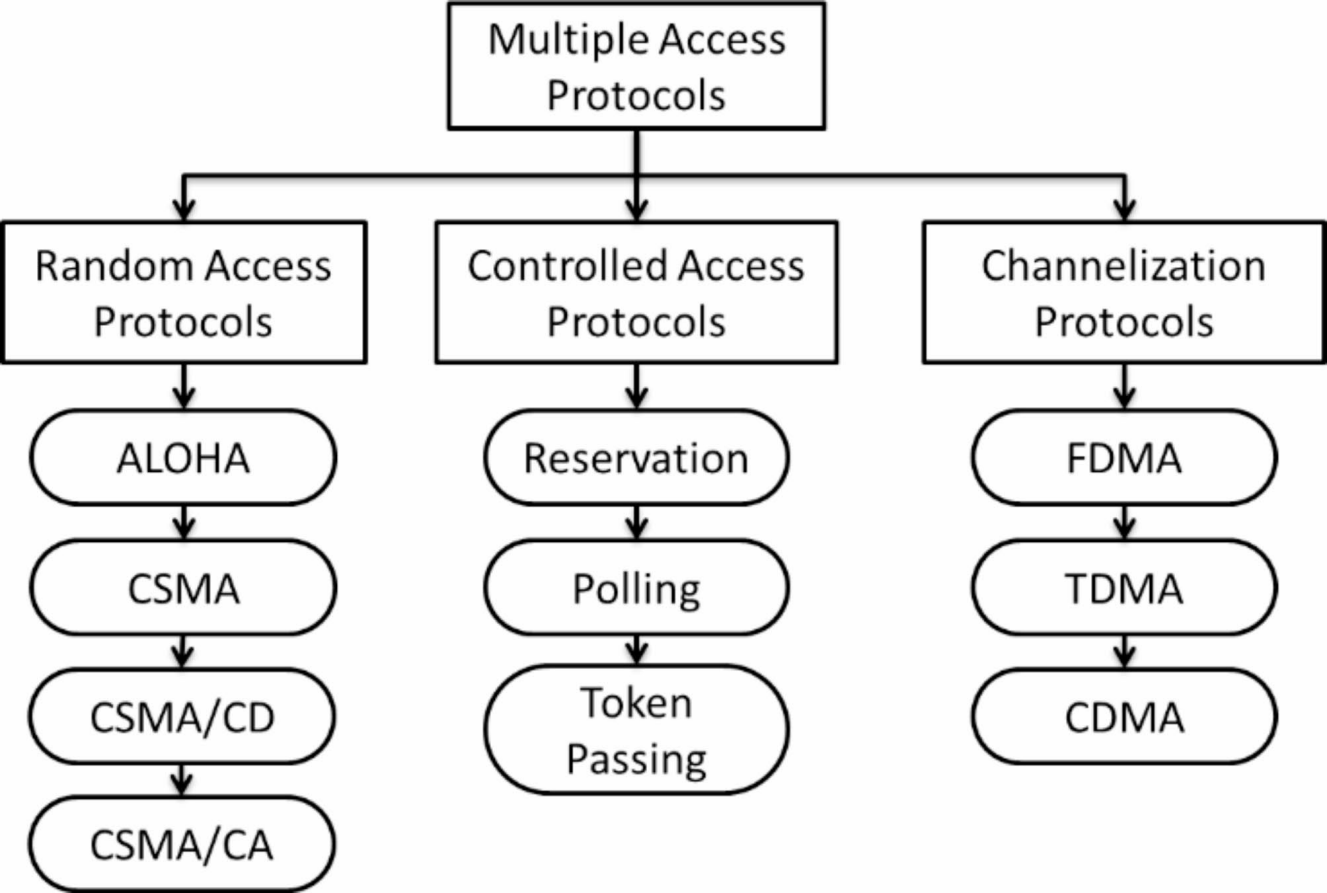
Classifications of MAC Protocol.

The data link control will function when a devoted connection between the dispatcher and the beneficiary. If not, numerous stations can approach the conduit at the same time. So, there is a chance of collision and crosstalk. Hence, various approaching procedures are mandatory to reduce interferences and prevent the superimposition of the data signal. Thus, proprieties are mandatory for allocating data on non-devoted stations. Manifold approaching procedures can be segmented and added as shown in Fig. 1.

## Random access protocol

This protocol exhibits the same superiority for all the stations that no place has extra essentials than the additional station. That means any station can transmit the data if the transmission channel is free. The transaction will be performed depending on the medium’s state. The stations in this protocol do not have a specific time for their transmission and no particular classification of stations. The Random-access protocols are more segmented into:

(a) ALOHA – this protocol is mainly used for non-wire local area networks but for public standards. In this channel, various stations can convey data simultaneously. Hence there is a possibility of interference and data being distorted.

Pure Aloha: In this, the position transmits data and holds it back for its credit. If the acknowledgment doesn’t originate in a prearranged period, then the station stands by for some volume of period again. That extra waiting period is named back-off time ( $\:{\text{T}}_{\text{b}}$) and resends the signal. As the stations are operated with different time periods for data transmission, the probability of occurring collisions and attacks is mitigated^43^.

Slotted Aloha: The slotted aloha is the same as the original aloha. But the difference is, here segregate the period into slots. And transfer the data only at a particular time period. If suppose, the particular time does not arrive, till that the data of that time slot wait. So that this method mitigates the interference.

(b) CSMA.

The CSMA (Carrier Sense Multiple Access) limits the number of interferences as the station is essential to intellect the channel for indolent/demanding formerly conveying data. If it is sluggish, it transmits data, or it holds back until the conduit develops indolent. Though, there is an opportunity for interference in CSMA as a result of dissemination interruption. Let us consider an illustration of station A and station B. Initially station A wants to send data. For that, the first step is the station to verify the channel for any inconvenience. Suppose it finds any attacks in the channel, the CSMA starts distributing the data to the channel^44^. However, the channel is to be conscious when the first bit of the data comes from stations A to B. Also, the CSMA will diagnose that the particular node is indolent and transmit the data. Thus, this method seriously affects the beginning stations A and B in terms of interferences.

(c) CSMA/CD.

Carrier Sense Multiple Access (CSMA) with Collision Detection (CD) tends to detect the collision if it occurs. Once the collision is detected, this scheme will terminate data transmission.

(d) CSMA/CA.

Carrier Sense Multiple Access (CSMA) with collision avoidance (CA). The development of interferences diagnosis includes the transmitter getting recognition data. Regarding CSMA/CA is an effective method when there is a single source of data transmitted. But if more than one signal, one signal is created on its own, and another is an interference signal. That refers to interference that has happened. To discriminate between these two cases, interference should have a maximum effect on the established signal. The CSMA/CA technique is not a wired network. Thereby, the method CSMA/CA is employed in the study^45^.

**Controlled access**:

If an information signal is transmitted by a station, which has to get approval from all other stations.

### Channelization

The spectrum of the frequency range of the channel is divided by period, frequency, and code to several stations to utilize the station at the same time.

Frequency Division Multiple Access (FDMA) – The accessible range of frequency is alienated into the same number of fields. Individually each station will operate under its peculiar specific frequency band. Guard bands are also incorporated because; two rounds will not get overlap. Hence, the problem of cross-talk and noise will be curtailed.

Time Division Multiple Access (TDMA) – The frequency range is divided among many stations. The interferences of the multiple stations can be prevented by allocating time slots for each station to send the data. Nevertheless, harmonization has a fixed cost as each station wants to identify its time slot. This is overwhelmed by accumulating harmonization bits to each space. An alternative problem with TDMA is dissemination delay which is determined by the accumulation of guard frequency ranges.

Code Division Multiple Access (CDMA) - One conduit conveys all communications concurrently. There is neither detachment of frequency range nor partition of time-period. For instance, if there are countless persons in a chamber all talking at a similar time-period, then flawless data response is also probable if only two individuals communicate with identical linguistics. Correspondingly, data from various stations can be spread instantaneously in different code languages. But then, on the contrary, the preservation material will upsurge the network overhead. The PLC system network topology fluctuates fast so that the regulator sachet interferences likelihood growths and the speed of time-slot conservation conjunction will sluggish down, distressing the network presentation^46,47^.

### Features of TP-LINK-based AC Wi-Fi kit

The wireless network has been extending its coverage to homes, offices, and places that the router’s wireless signal cannot reach. This application is possible when the modem TP-LINK Gbps passes through the power cable Wi-Fi kit. The power line adapter is required, and the power line connecter involves two devices; one is a TP-LINK TL-WPA8630P stretcher, and the other is a TL-PA8010P adapter. The modified Homeplug AV2 method means that the modem TL-LINK provisions MINO beamforming technology. The modified modem provides efficient throughput depending on the environment; for example, the physical layer has a throughput of 1200 Mbps. In addition to that, the modified TP-LINK modem supports all types of contemporary wireless networking values capable of 802.11 ac. The designed adapter is a kind of double-group maneuver that processed to an entire supreme imaginary bandwidth of 1234 Mbps. The previously used TL-PA8010P converter has one ethernet port for linking the adapter with the wireless router. But the modified modem TP-WPA8630P protractor has three Ethernet docks for involving up to three strategies in the network. When the TP-LINK enlarger adapter is instigated on a HomePlug AV2 description, the MIMO signal feasibility enhances the data transmission speed, significantly highly attenuated channels. Thus, MINO enables the transmission on any dual-line couples inside a trio-line PEN conformation comprising line, unbiased, and defensive terrain. In addition to that, the modified TP-LINK adapter contains an Auto-sync option that can add an extra TP-LINK enlarger to the PLC grid, and the synchronous option makes position including SSID, keyword, or preparation for the entire strategies that develop the PLC network. It is probable to fix the modified modem’s package tpPLC, the portable request for Android and iOS on the smartphone. The software maintains the power line communication web and all the campaigns, such as data transmission speed adjustment, prior arrangement, and supervising stretcher.

The Wi-Fi expansion would help achieve internet connectivity in other areas such as homes, offices, and buildings employing TL-WPA8630P PLC adapters. Also, spread this linkage with different wired and wireless strategies. Another significant advantage of having an integrated grid is firm network congruence regardless of whether devices are moved in a specified area. The proposed combined web harnessed for seamless video streaming; this connection accomplished stable connectivity while online betting and net surfing on audiovisual aid transportable campaigns in inapproachable places in the remote household. As stated above, one modem enlarger will support three local area network acquaintances to LAN. Likewise, the mobile applications of tpPLC Utility and tpPLC App on iOS permit control over all devices in the home. This will help manage all the objects in the home or office and protect this network. The parameter of transfer rate measures the efficiency of the PLC adapter. The data transmission rate determines network connectivity.

**Fig. 2:**
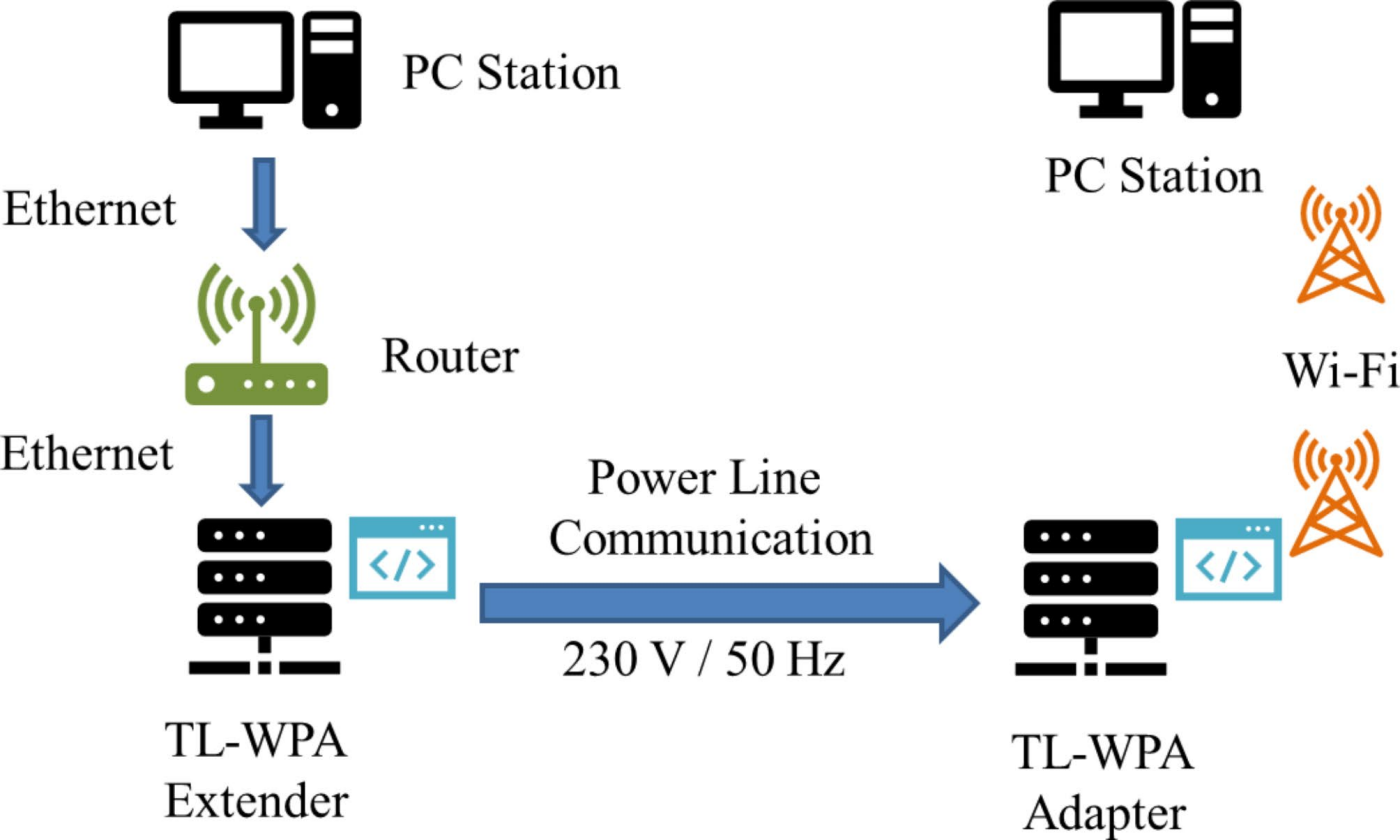
General diagram of workstation using TP-LINK.

Figure 2 displays the construction of wiring in a home that exploits plug-andplay capability with HomePlug AV2 specification. If suppose the connection is between both PCs directly, the occurred transfer rate is below 900 Mbps which is drastically low. This is done without a BPL adapter. Hence this drawback makes a restriction for BPL adapters.

### Throughput of different wireless network technology

**Fig. 3:**
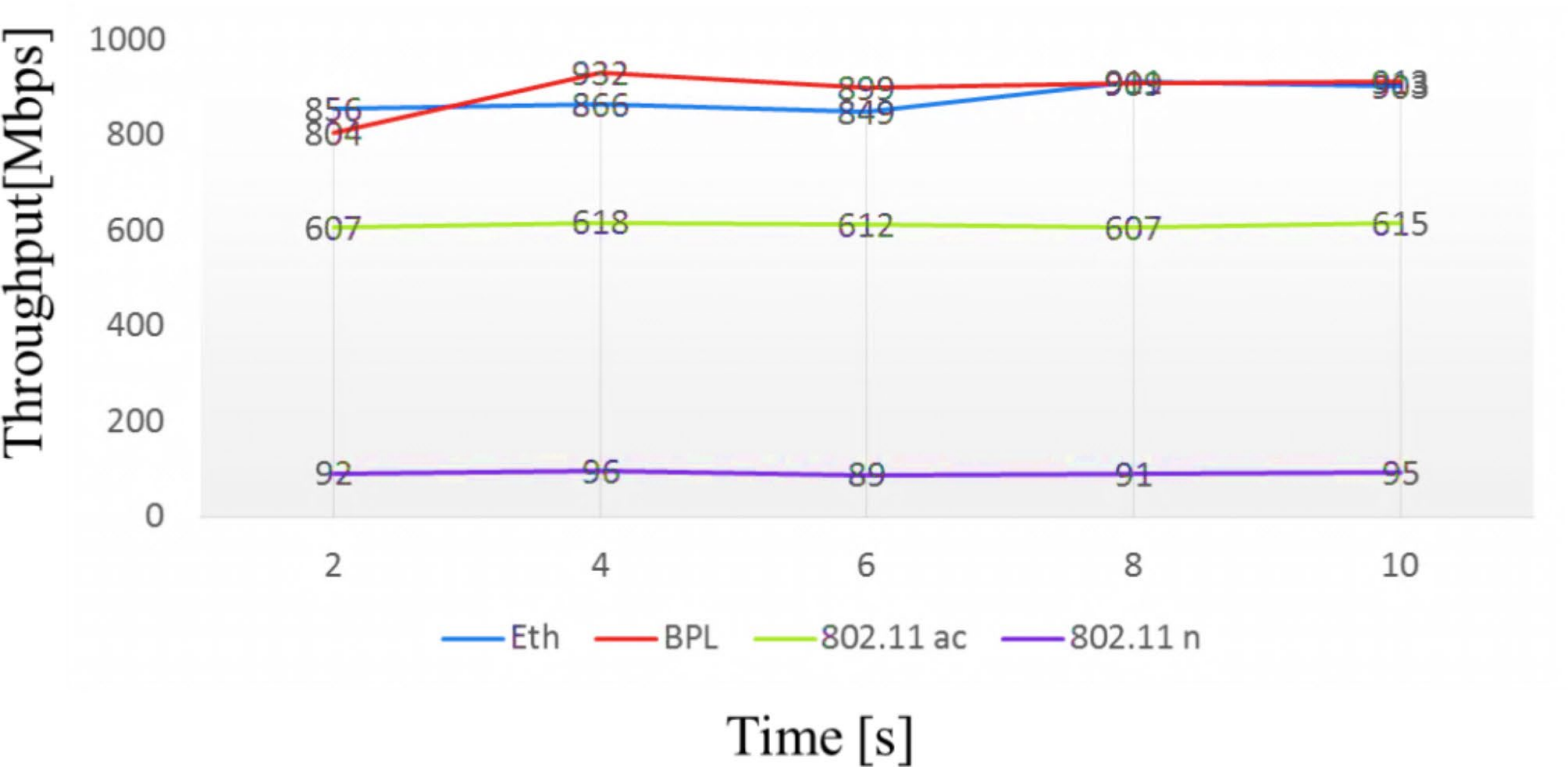
Comparison of observed throughput [Mbps] vs. Time [s].

The technical term throughput of one particular network is the amount of data displaced from one place to another in a specified time, and it is measured in bits per second. The comparison of the throughput of Ethernet communication channels between computers is illustrated in Fig. 2. We have taken various network topologies like IEEE 802.11 AC typical, IEEE 802.11 n router, Ethernet, and Broadband Power Line (BPL). Initially, the router runs on IEEE 802.11 AC typical and notes the performance. The IEEE 802.11 provides a throughput rate not exceeding 600 Mbps. Next, the most frequently utilized IEEE 802.11n router is evaluated, and it delivers a throughput of not more than 100 Mbps. The data transfer rate of the Broadband Power Line adapter technology majorly is contingent on the inner construction of the computer and the connection of each device to the electric power network owing to system electric resistance transformation. The Ethernet router connection between two computers provides a throughput of 900 Mbps. From Fig. 3, the wireless technology with power line communication gives maximum throughput of 900 Mbps.

### Performance of data transfer rate

**Fig. 4:**
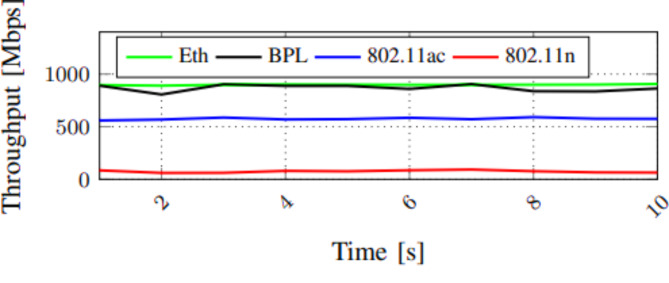
Measurement of transfer rate related to the time.

The network’s bandwidth defines the maximum amount of data transferred per second on a link. The frequency range is generally determined in bits per second, Mbit per second, or Gbit per second. The data transfer rate is related to the speed of data transformation from one place to another. Otherwise, it refers to the data transfer speed between a peripheral device and the computer. It is conventionally computed in Megabits per second (Mbps) or Megabytes per second (Mbps). The data transfer rate depends on speed, but it gets affected by the sender or receiver. There are also various technologies compared, such as IEEE 802.11 AC typical, IEEE 802.11 n router, LAN, and Broadband Power Line (BPL). Initially, the router runs on IEEE 802.11 ac typical and notes the performance. The IEEE 802.11 provides a throughput rate not exceeding 700 Mbps. Next, the most commonly used IEEE 802.11n router is undergone evaluation, and it delivers a throughput of not more than 400 Mbps. The data transfer rate of the Broadband Power Line adapter technology is mainly based on the computer’s core construction and the connection of each device to the electric supremacy system because of system resistivity variation. The Ethernet router connection between two computers provides a throughput of 1000 Mbps. From Fig. 3, the wireless technology with power line communication gives maximum throughput of 1200 Mbps. From Fig. 4, the wireless technology with power line communication gives maximum throughput of 1200 Mbps.

### Average delay of the network

**Fig. 5:**
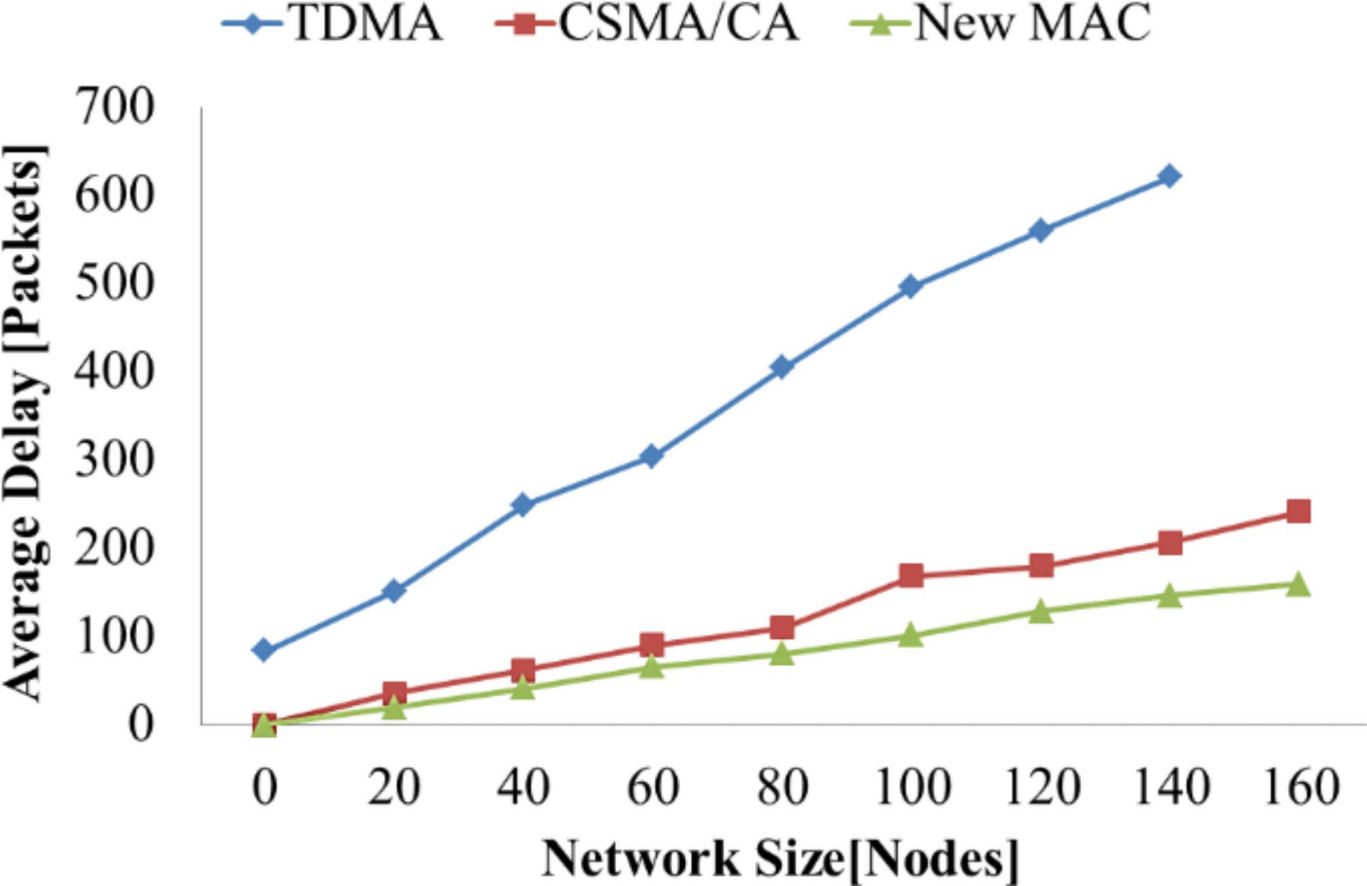
Average transmission delay under various network sizes.

The average delay is used to evaluate the network transmission efficiency. The evaluation outcome taught the system the impact of a modified BPL adapter. The graphical analysis of the average delay to the network size is depicted in Fig. 5. The definition of the expected time delay of the grid is the mean period taken for a system to magnificently communicate a data sachet to the size or distance of the data sachet interval. The typical time delay is an excellent parameter for investigating the proposed protocol’s transmission efficiency standard. This analysis computes the typical communication time delay of the system with supreme network consumption beneath the different network dimensions. For effective network utilization, 10 different network sizes have been selected. Also, we set the communication rate as 20 kb/s, and the span of a sachet is 27 bytes. The software recreation result is taken for 20,000 sachets after being diffused magnificently. When the network transmission efficiency is attained maximum, the average transmission delay is observed in Fig. 5. It can be noted that the modified BPL adapter has experienced minimum typical communication time delay and advanced proficiency as the network size develops.

### The performance analysis of network utilization (or) Stability

**Fig. 6:**
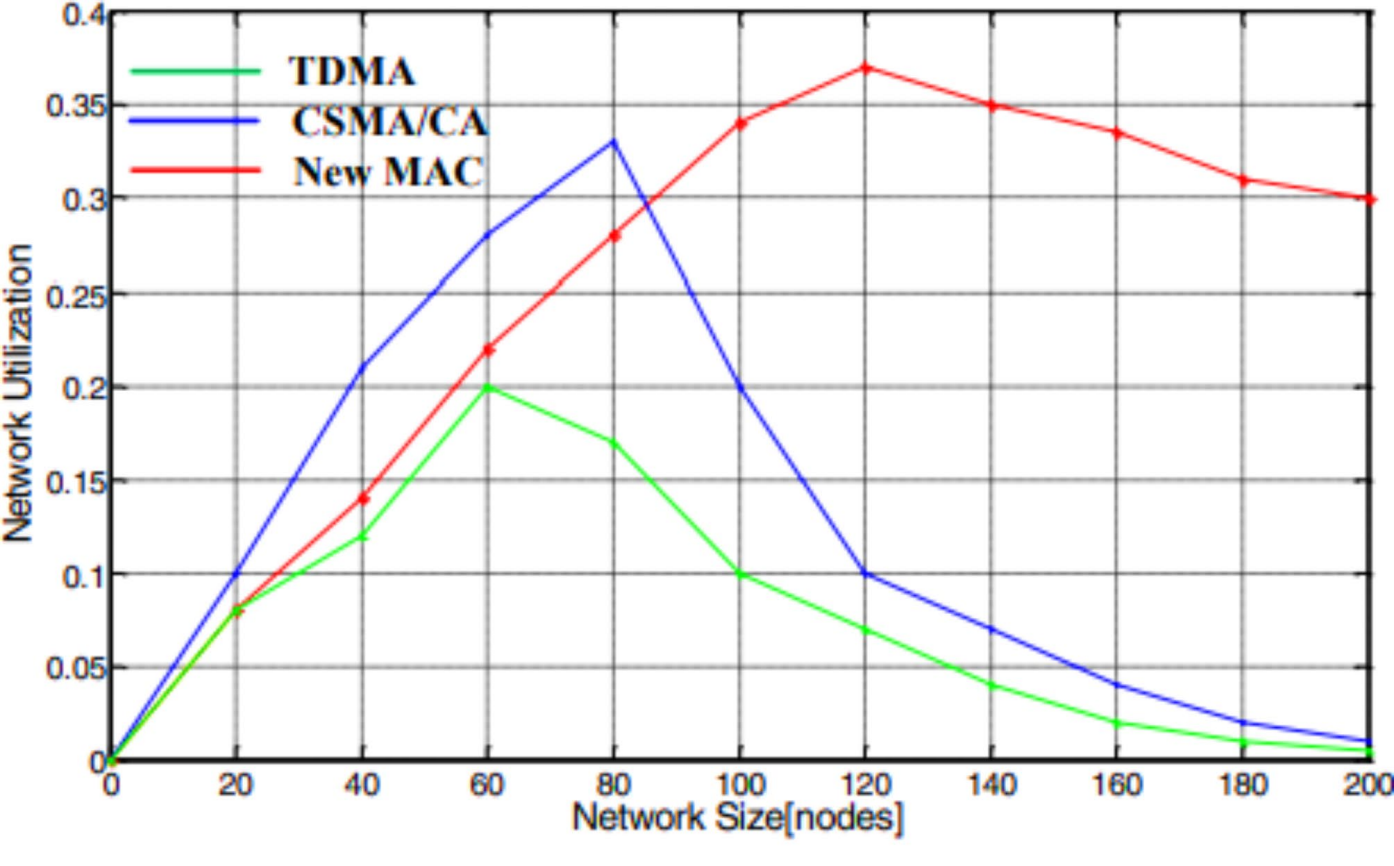
Comparison of various protocols based on network utilization.

Based on the utilization of the network, the stability of the technology has the ability, as shown in Fig. 6. The network stability of a network is a correlated relationship between network utilization and network stability. The determination of network stability of four network technologies, like IEEE 802.11 ac typical, IEEE 802.11 n router, Ethernet, and Broadband Power Line (BPL), is shown in Fig. 6. From the results, if the size of the network is small, the network utilization is to some extent lesser than the other two network topologies owing to the decorum operating cost. Nevertheless, as the network size remains to develop, the network employment of other wireless technology except BPL fell compared to the slight decreases of the modified BPL protocol.

## Intra-cluster MAC protocol

Wireless communication is inevitable and blooming technology for various significant uses including catastrophe administration, martial, and sanctuary. However, the efficiency of the wireless communication network is based on specific constraints like energy, storage, and computation of the available resources. Hence, there is a requirement to develop resource-aware procedures and administration performances. This work proposes an effective mountable, and crash-unrestricted MAC protocol for wireless communication grids. This proposed protocol is based on stint-founded adjudication of the network standard to curtail the signal intrusion in transmitting data in the channel^18^. The outcome of the proposed intra-cluster MAC protocol is better in terms of energy consumption and scalability. The energy consumption is attained with the help of keeping the active mode to sleep mode while not in use condition. The scalability factor is achieved by segregating wireless networks as clusters and operating with them. Therefore, in this work, we proposed an energy-efficient MAC protocol, it is robust for inter-cluster impacts and prevents data packet drop due to the buffer size boundaries.

### General architecture of wireless network

Wireless communication is inevitable and blooming technology for various significant uses, including catastrophe administration, martial, and sanctuary. However, the efficiency of the wireless communication network is based on specific constraints like energy, storage, and computation of the available resources. Hence, there is a requirement to develop resource-aware procedures and administration performances. This work proposes an effective mountable and crash-unrestricted MAC protocol for wireless communication grids. This proposed protocol is based on stint-founded adjudication of the network standard to curtail the signal intrusion in transmitting data in the channel^18^. The outcome of the proposed intra-cluster MAC protocol is better in terms of energy consumption and scalability. The energy consumption is attained with the help of keeping the active mode to sleep mode while not in use condition. The scalability factor is achieved by segregating wireless networks as clusters and operating with them. Therefore, in this work, we proposed an energy-efficient MAC protocol it is robust for inter-cluster impacts and prevents data packet drop due to the buffer size boundaries.

### General architecture of wireless network

**Fig. 7:**
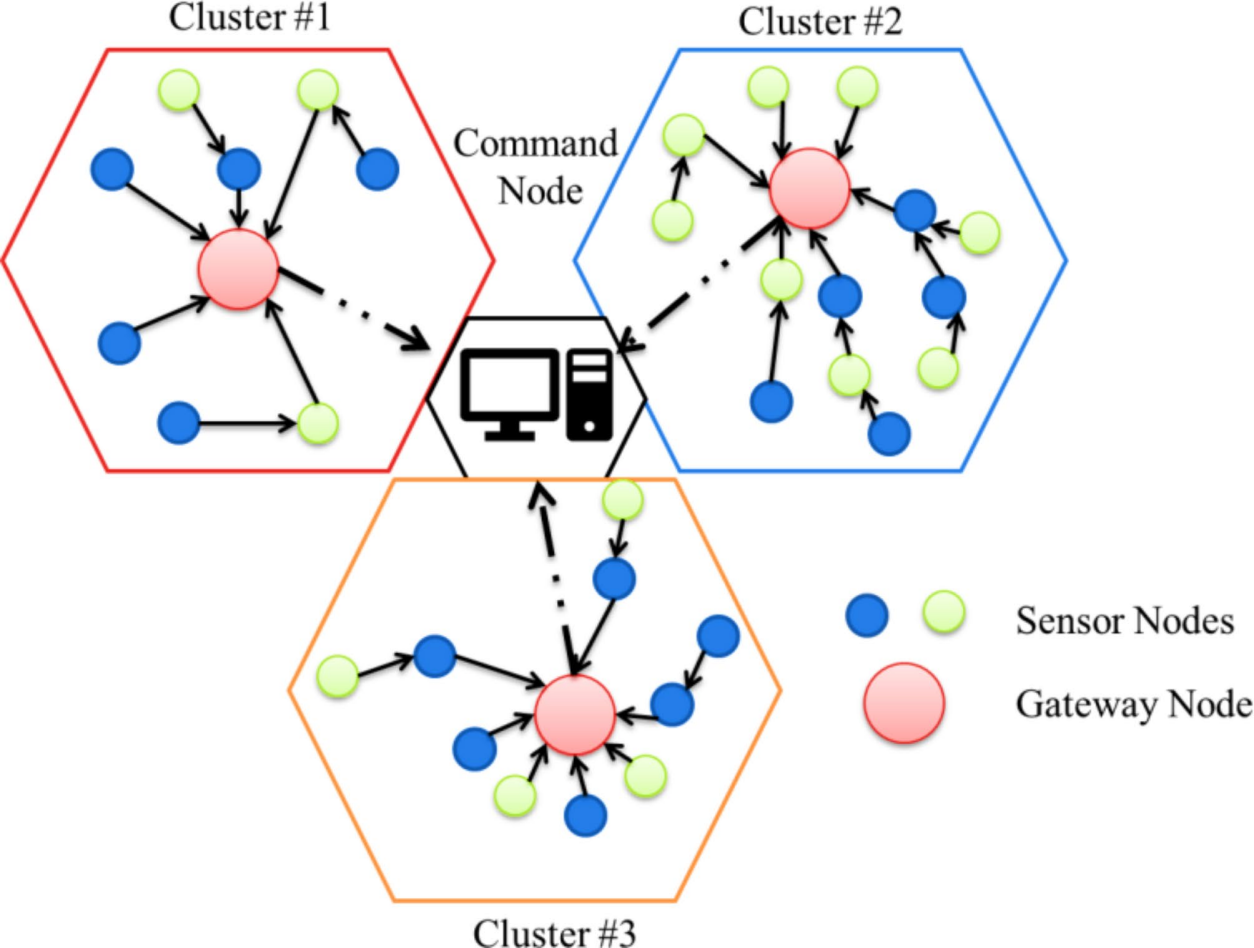
The architecture of the clustered network.

The conventional construction of a wireless network is illustrated in Fig. 3. In this network, the number of nodes that can be grouped is called a cluster, and a single command node can control the cluster. There will be a gateway node among the clusters responsible for managing all the clusters. All the clusters are operated by command nodes or by gateways. Through long communication links, the gateways communicate with the command node in a wireless network. The receiver gets the commands and sends the data to their gateway node. The gateway node processed the received data and retransmitted the fused information to the command node. Since gateways are significantly less energy constraint, which play an important role in sending and receiving the data. Also, the gateway fixes multi-hop routes based on the current state of the network and sends route updates to the nodes. Depending on the route formation, some nodes should have acted as relays. By doing so, the nodes can transmit power based on their next-hop neighbor. If the next node is situated nearby, the node spends less energy communicating. Perhaps, the next node is located far away; it consumes some more power to go there. The common architecture of clustered networks is shown in Fig. 7.

### Synchronization mechanism

The communication medium of a network is required to have time-based access. So that all the communicating nodes will be harmonized. The clock drift is an undesirable effect that can create interferences among the transmission nodes. To maintain synchronization in the nodes, periodic enforcement has been used. The irregular enforcement has adjusted the clocks to the reference value. The adjustment frequency is based on many parameters, such as the schedule and the clock drift time. The proposed work maintains the harmonized clocks because of linking the GPU receiver with gateway nodes. The accurate reading clocks in the gateway nodes are responsible for harmonizing the sensors in a group of nodes. Utilizing broadcasting the harmonization messages to the nodes in a cluster, the other nodes are alerted when they are expected to have a collision. For effective synchronization, the network requires a period-opening setting up.

### Time period -slot Scheduling

This session’s time period-slot arrangement mechanism is a sub-set of rerouting an arbitration phase. The transmission schedule has been planned among the gateway of the network in a group of nodes. However, slot scheduling brings a multi-hop traffic problem due to the data traffic. Thus, DFS (Depth-First Search) and BFS (Breadth-First Search) techniques will set transmission slots. However, the DFS algorithm consumes a lot of energy due to a large number of communications, and the BFS algorithm leads to a packet drop because of the size of the buffer. Thus, we need to propose a new time-slot mechanism called the Intra-cluster slot allocation algorithm. The Tabu-based simplification methodology inspires that. This protocol maximizes network performance through dynamic channel allocation and selection, allowing devices to switch to optimal channels based on current network conditions and interference levels. It employs algorithms which assesses quality of the channel and assign channels to devices accordingly by minimizing congestion. The protocol prioritizes traffic based on application needs, ensuring that critical applications receive the necessary bandwidth and low latency. This approach enhances the overall efficiency and reliability of communication in a smart home network. The target of Tabu based simplification method is to avoid the irrelevant power disbursed among idle nodes. The futile transmission has been performed among active and sleep nodes.

The proposed protocol has the potential to manage N number of connected devices in an automated home environment with absolute efficiency and data rate.

Adaptive time slot allocation and clustering approaches achieve the effective communication by the segregation of Contention-Free and Contention Periods.

More optimized data rates are then achieved since devices can always evade the crowded frequencies with the dynamic channel hopping proposed. Further, clustering supports localized communication management, therefore reducing the burden on central networking, and in combination assures that this protocol can scale with efficiency and maintain dependable communication amidst extensive device diversity.

By assigning time slots to devices, it controls communication, reduces the likelihood of collisions, and keeps an eye on channel conditions. It prioritizes data traffic according to preset standards, making it easier to send important information on time. The coordinator dynamically reallocates time slots to make room for new devices without interfering with ongoing communication. In order for new devices to register and integrate into the network. In order to preserve stability and efficiency, the coordinator also rearranges the time slots when devices are taken out.

### Intra-cluster collision avoidance

The collision can occur when a specified node receives multiple data at the same period. The TDMA method provides no collision among the nodes in the same cluster. Collision avoidance is achieved, because of selecting a node and scheduling the same node to transfer the data to a particular period. Nevertheless, the process of various groups is followed simultaneously, and intra-cluster collisions cannot be governed. The intra-group network prevents the features of the different slots assigned within each cluster. Thereby, mitigating the network performance of intra-group interference. The collision among internal network nodes can be reduced by fixing some frequency bands between them. Further, unique ranges also will be provided for each group of nodes. But this procedure will consume a lot of energy since the active time of a node is maximum.

One way to prevent an intra-cluster collision is to assign a different time slot for the adjacent gateway of the other cluster. This precautionary operation needs to know the time slot for the adjacent gateway of the different clusters. However, to avoid group interference problems, not only the detail of the schedule of an adjacent node is important. To solve the interference problem completely, the time slot or period of every node in a cluster as well as the neighboring cluster nodes should have been known. If suppose the group network is extensive, there will be more comparison required. And based on the comparison results, the modification or elimination of periods of the gateways would reduce the collision. The NP-hard problem determines the appropriate rearrangement of the time slot. Sometimes the modification of scheduling time slots also will induce the collision. Hence, until we obtain a better solution, the comparison must be done continuously. In addition to that, modifying the schedules will affect the efficient energy consumption of the network.

Hence this work proposed a new intra-cluster protocol as follows. The first step of the protocol is to compute the transmission schedule for the nodes within their related clusters. Then choose a primary (head) gateway from a set of primary gateways. The primary (head) gateway is elected through a round-robin fashion. For each arbitration phase, there will be an ahead gateway. The primary (head) gateway is used to store the transmission period of all the clusters. And then the programs are combined to determine any powerful inter and intra-cluster collision. Suppose, if any collision is detected, the head gateway verifies the fake tree of nodes and reallocates the affected tree with the standard tree structure. The swapping of trees in this context is known as a tree swap. In this manner, the proposed intra-cluster MAC protocol benefits the wireless network for the energy-efficient scheme.

The protocol mitigates interference and collisions in power-line communication, allowing devices to transmit only during assigned slots, and employing channel hopping to avoid congested channels. If collisions occur, the protocol detects them and initiates a retransmission strategy after a random backoff period. To handle network congestion, it classifies data packets by priority, ensuring critical traffic is transmitted first. Dynamic time slot allocation allows high-priority data to receive more bandwidth during peak times.

### Performance of intra-cluster protocol

**Table 1:** Performance analysis of the proposed intra-cluster protocol.

Parameters	Intra-cluster Protocol
Energy Consumed (Joules)	0.018
Average delay/packet (sec)	2
Throughput	0.94

Table 1 summarizes the performance analysis of the proposed intra-cluster MAC protocol. The performance of the proposed MAC protocol can be evaluated with different parameters such as energy consumption, the average delay per packet, and throughput. First, the value of energy consumed by each node in a network during transition is taken. Next, the average delay evaluates the system’s communication competence. The typical time-period delay of the system is the meantime taken for a web to effectively convey a data sachet to the size or dimension of the data sachet interval. Therefore, the average delay is an excellent parameter for investigating the proposed protocol’s transmission efficiency standard. This analysis computes the mean communication time delay of the grid with supreme system consumption beneath the diverse magnitudes of the network. The technical term throughput of one particular network is the amount of data displaced from one place to another in a specified period, and it is measured in bits per second.

## Architecture of power line communication

### Network structure

**Fig. 8:**
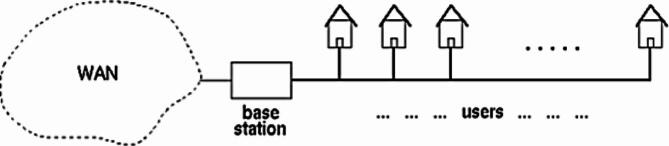
General architecture of the PLC system.

Power Line Communication Networks connected various users/devices to the backbone communication network such as LAN and WAN through base station/PLC Modem as shown in Fig. 8. The PLC system can be employed in telecommunication access networks covering 32 MHz. It acts as a feeler fabricating electrostatic energy, originating interferences and instabilities in the wire data transmission services within the same recurrence band. The small energy source systems have numerous grid network topologies consuming tree construction^48^. There are many network access points from the transformer module to the users/devices, constituting various network topologies. The specific PLC network topology is accumulated with the help of a base station that makes communication links between users of the PLC network and WAN.

Let’s consider that the interior transmission amid customers of the PLC system is carried out by the main post (i.e.) in this context PLC modem. Therefore, two types of transmission directions are achieved in the interior of a PLC system:

- Downlink – it refers to the communication of the central station to the system customers.
- Uplink refers to communication since the system users to the main station.

The transmitted signal is referred from the main station to all the customers in the system during the downlink management. Similarly, the transmitted signal from an employer to the main station is reached via all additional customers in the network system during uploading direction. That means the PLC admittance system embraces a reasonable bus construction regardless of the circumstance that the little-power supply grids have topographic tree anatomy^2^. It is effective if we deliberate the PLC network system segment or any other portion of the PLC network. On account of that, we contemplate the PLC network construction as a national bus system.

### Transmission system

The PLC network’s transmission system mainly depends on multiplex and modulation schemes for their applications. Primarily used the modulation types of CDM – Code Division Multiplexing and OFDM – Orthogonal Frequency Division Multiplexing schemes^49^. An OFDM constructed on a communication system can provide a numeral of channels separated in the regularity band, as designated in^50^. In the context of CDM founded transmission system, the entire existing communication field is separated by extraneous codes. All the subcarrier signals of the OFDM can be used for transmission with the help of the TDMA schemes.

In general, the concept of above-all data communication shame is that the transmission channels have been alienated in the recurrence rate, in terms of time or code range. So, we can accomplish that the PLC communication systems appear to have a rational conduit construction self-governing of the employed communication expertise. Consequently, in the progress of the MAC sheet, it is probable to pact with reasonable canals which are succeeded by a MAC etiquette. The rational communication conduits have additional import for each measured communication technique, then the inquiries, completed on a moderate level, can be employed in any of the broadcasting outlines.

### Near-field communication

The NFC technology is the short-range low data rate wireless communication technology that operates at a 13.56 MHz frequency. The devices in the technology can communicate in contactless mode^51^. The contactless mode of communication has been performed by employing electromagnetic waves. This technology permits two devices connects NFC chip to communicate for various applications.

**Fig. 9:**
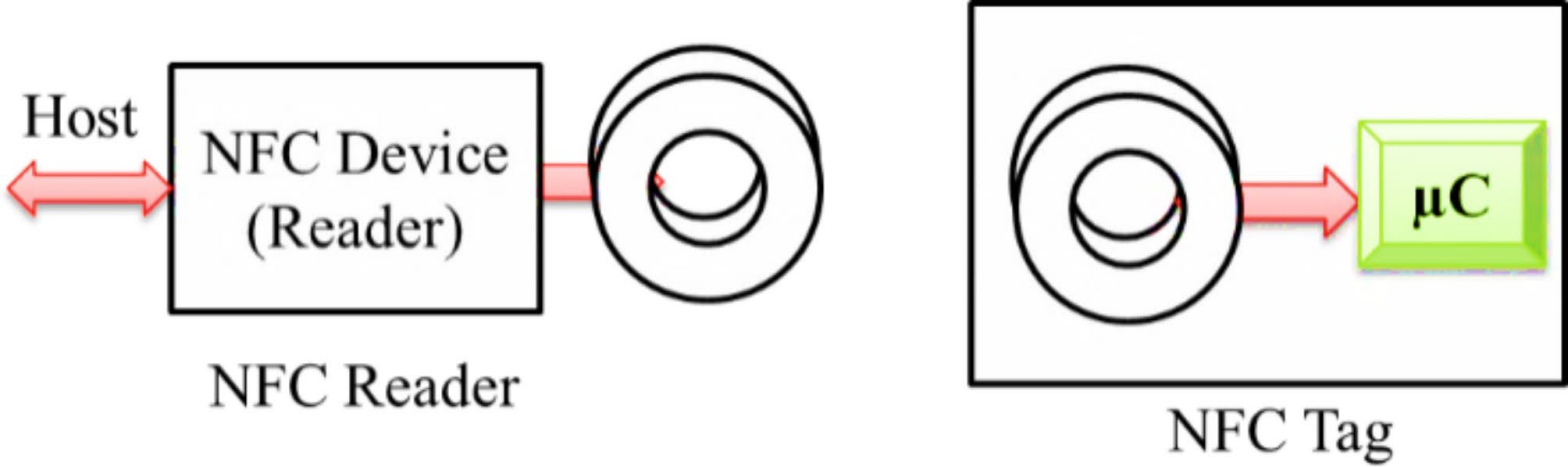
The conventional model of the NFC Network.

The conventional model of the NFC network is illustrated in Fig. 9. The NFC network consists of two devices: initiator and target devices. The NFC tag can act either as an active or passive device. However, an NFC reader is always a functional device. These NFC devices can operate in active-active mode or active-passive modes. Both NFC devices exhibit their power in active-active ways. In the case of the active-passive method, the passive device grasps its power from receiving electromagnetic waves from the active devices. In smart home design, the NFC tag connects two components and shares the information through them.

The proposed work outlines several scientific measures to secure the smart home network against unauthorized access through integrated NFC technology. Among other measures include using strong encryption techniques in transmitting data over the network so that any potential interceptor cannot read it in any useful format. This is however backed by stringent authentication mechanisms which guarantee that limited operational range plus extra layers of security implemented within the NFC only allows authorized devices to connect to the network. All these, therefore, demand physical proximity for communication. It further comprises software updates on vulnerability remediation and related product updates.

The overall power consumption of the systems is reduced by enabling adaptive channel selection, minimizing data collisions and organizing devices into clusters for effective Communication. NFC provides several advantages such as enhanced security due to its short-range operation, low power usage and ease of use. These factors make NFC suitable for specific applications but may restrict its use compared to other communication technologies.

## Modified intra-cluster MAC protocol

**Fig. 10:**
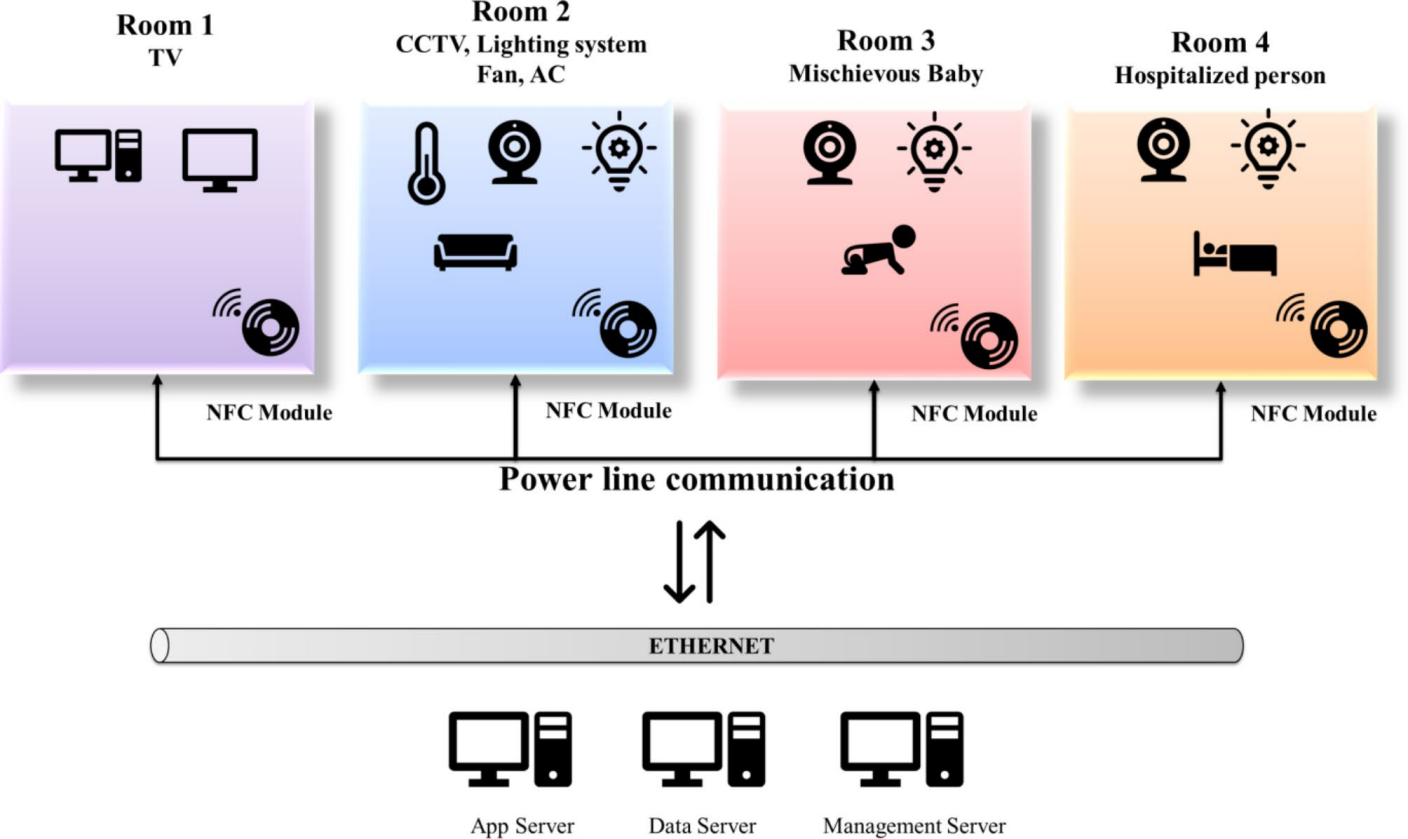
System structure of PLC Network.

In this session, a novel modified Intra-cluster MAC protocol was proposed and designed for Efficient Energy consumption. The conventional construction of the topology is exposed in Fig. 10.

### Proposed prototype

Smart homes are assumed to be a new trend to yield valuable services for the customers to reduce cost or energy and create a comfortable life. The smart home has the capacity of automatic control over the home appliances by turning off the devices during the absence of the user. Thereby the smart home application curtails the unnecessary usage of power. The smart home has the feature of automatic operation, and hence our life has become more accessible. Wireless communication engineering is presently the leading adapted technology for innovative home applications in state-of-the-art methods. But the wireless technology has its issues while signal spreading and exposure limits over a greater area. So, the requirement for another replacement technology is desired. Nowadays, Power Line Communication is getting attention for constructing broadband networks which bring increased data transfer rates based on power grids. Also, power line communication has salient features over wireless technology for smart home automation, such as widening the network area. Moreover, there was no problem when the wall worked with conductive material. Hence, incorporating wireless technology with power line communication brought more exciting advantages to the innovative home design. Therefore, this work combined the properties of power line communication and MAC (Wi-Fi) protocol in this work. In the existing system, the researchers used power line communication with Wi-Fi or power line communication with ZigBee.

Also, every device of the smart home can be linked with the use of Near Field Communication (NFC) technology. NFC in smart home networks faces security vulnerabilities like eavesdropping and relay attacks. The proposed MAC protocol addresses these by implementing encryption and authentication mechanisms to secure communications. It also ensures backward compatibility with existing power-line and NFC devices, facilitating seamless integration in the smart home environment.

**Fig. 11:**
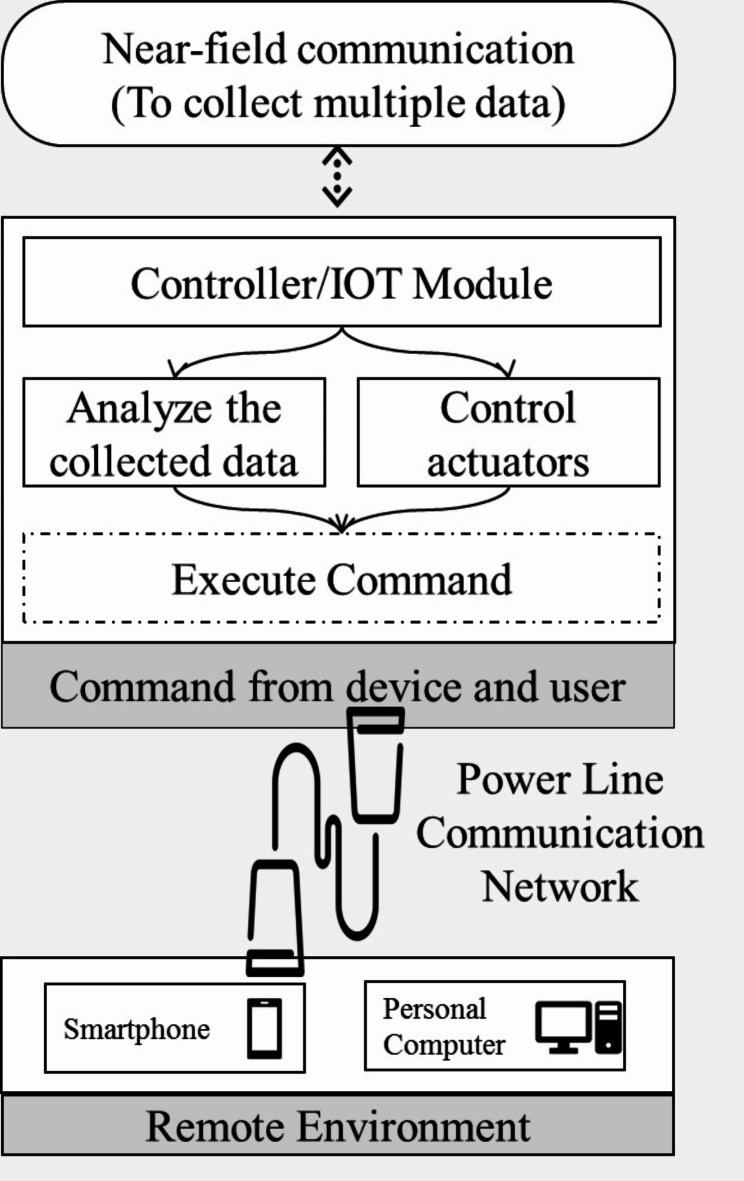
General architecture of the proposed prototype.

Figure 11 describes the general architecture of the proposed ideas. The proposed research work involves the design and implementation of a Modified MAC protocol for enhanced secure communication.

**Fig. 12:**
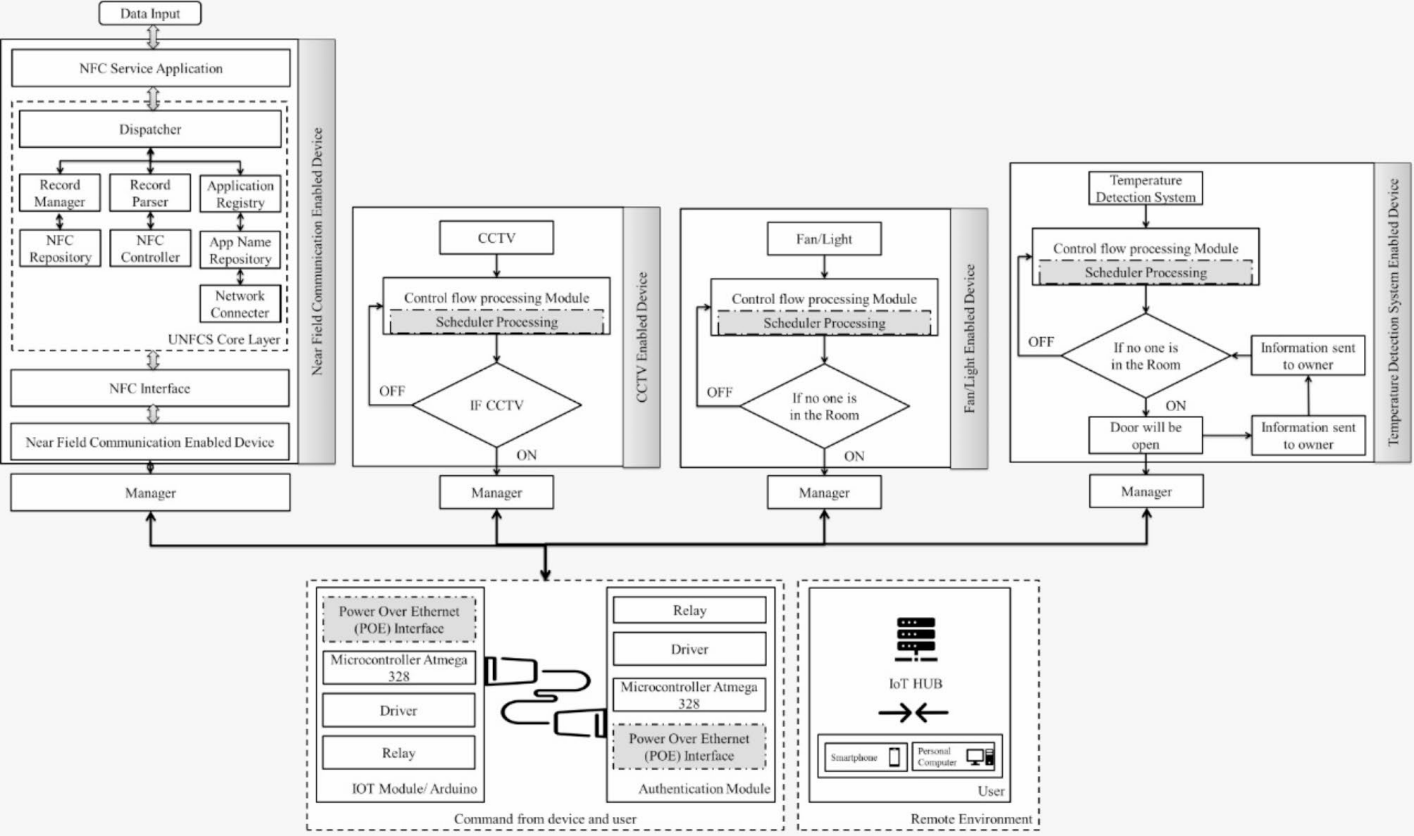
Overall structure of the smart home environment.

The entire design structure of the proposed work has been explained in Fig. 12. The overall design involves the controlling actions of the fan, light, CCTV, and temperature sensor in a smart home. The details of the particular applications have been sent to the respective authority persons. Detailed design procedures are also given in the design flow.

### Protocol design

**Fig. 13:**
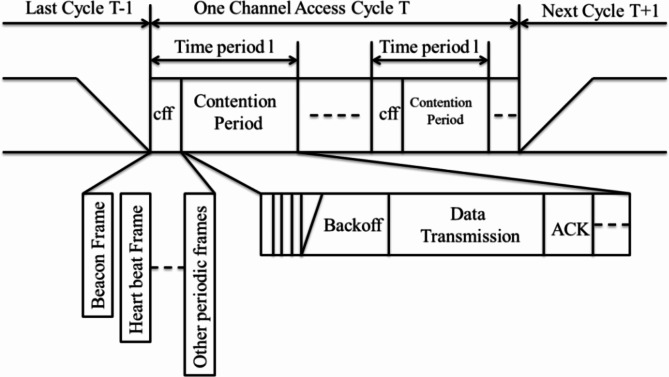
Periods of the modified intra-cluster MAC protocol.

The modified Intra-cluster MAC algorithm segregates the communication conduit admittance period into diverse time period, which the primary node or primary node can control. Every time period is separated as a Content Free Period (CFP) and a Contention Period (CP). In addition, the disagreement range is separated into four sessions: significance determination, random backoff, data transmission, and ACK response. The time arrangement illustration of the procedure is revealed in Fig. 13.

The Contention Free Period is allocated solitary for limited nodes to interchange their data as irregular edges in classification such as beacon frame, heartbeat frame, etc. But, the local nodes of their time slot can access the Contention Free Period (CFP) and other local nodes’ time slots. The flexibility of the accessing property of the CFP is performed with the importance-based CSMA/CA technique. In the Contention Period (CP) all nodes can contact the same network. Precisely, the beacon frame and other timely frames directed in the current time period have the maximum significance, confirming the chance for the nodes to enter the networks impartially and promising the real-time necessity for the announcement. The algorithm is separated into five fragments: time period sharing, importance-based determination, backoff window fixing, ACK retaliation technique, and redeliver technique.

### Proposed protocol steps

The proposed MAC protocol consists of phases executed in proper time intervals. The sub-section discusses the different types of stages in the proposed mechanism. The next part describes medium access arbitration and clock synchronization. The following explains the steps of the proposed mechanism:

- *Data Phase*: The nodes in a network can collect the important data of a node. Also, the collected information can be reached into gateway nodes. The data transfer from the source node to the gateway node is via next-hop adjacent nodes.
- *Redirect and Adjudication Phase*: There are two modules; one is the redirecting sector, and the other one is the arbitration sector. The reroute segment is based on a gateway that determines the new route to the nodes in a group based on the assignment goal. Similarly, the adjudication sector fixed a time slot for a distinct node in a group of networks. This stage ensures interference-free data transmission on new routes in a network.
- ***Broadcast Phase***: This phase is continuously implemented with the help of gateway nodes. The responsible of the gateway node is to provide new routes, different periods, and commands for the next cycle nodes.
- ***Synchronization Phase***: This phase conveys the clock information to the nodes in a cluster by sending synchronization messages.

### Time period allocation

**Fig. 14:**
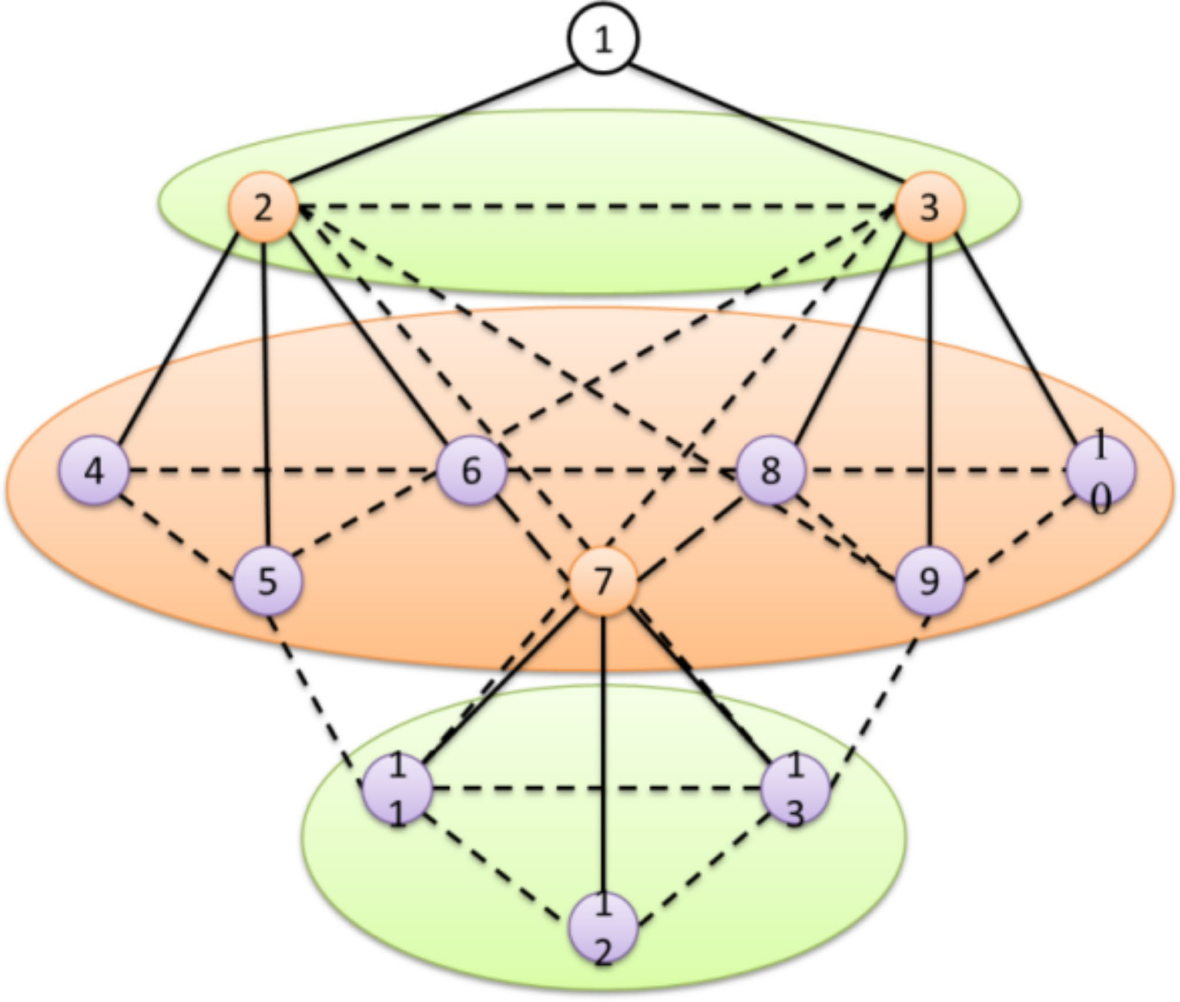
Logical topology of PLC network.

**Fig. 15:**
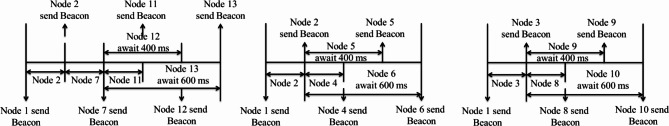
Time arrangement of beacon broadcasting in a beacon period.

Time duration has been separated with the help of routing methodology and beacons. The node is in the active position of the system entree, denoted as the primary direction of the time period. Here in this Fig. 14, the focus of the secondary node to the primary node is the original route. The topology of the main route in Fig. 15 indicates a solid black line. The core route follows the nature of the non-overlapping cluster methodology. Moreover, the time period of the main route depends on their clusters.

**Fig. 16:**
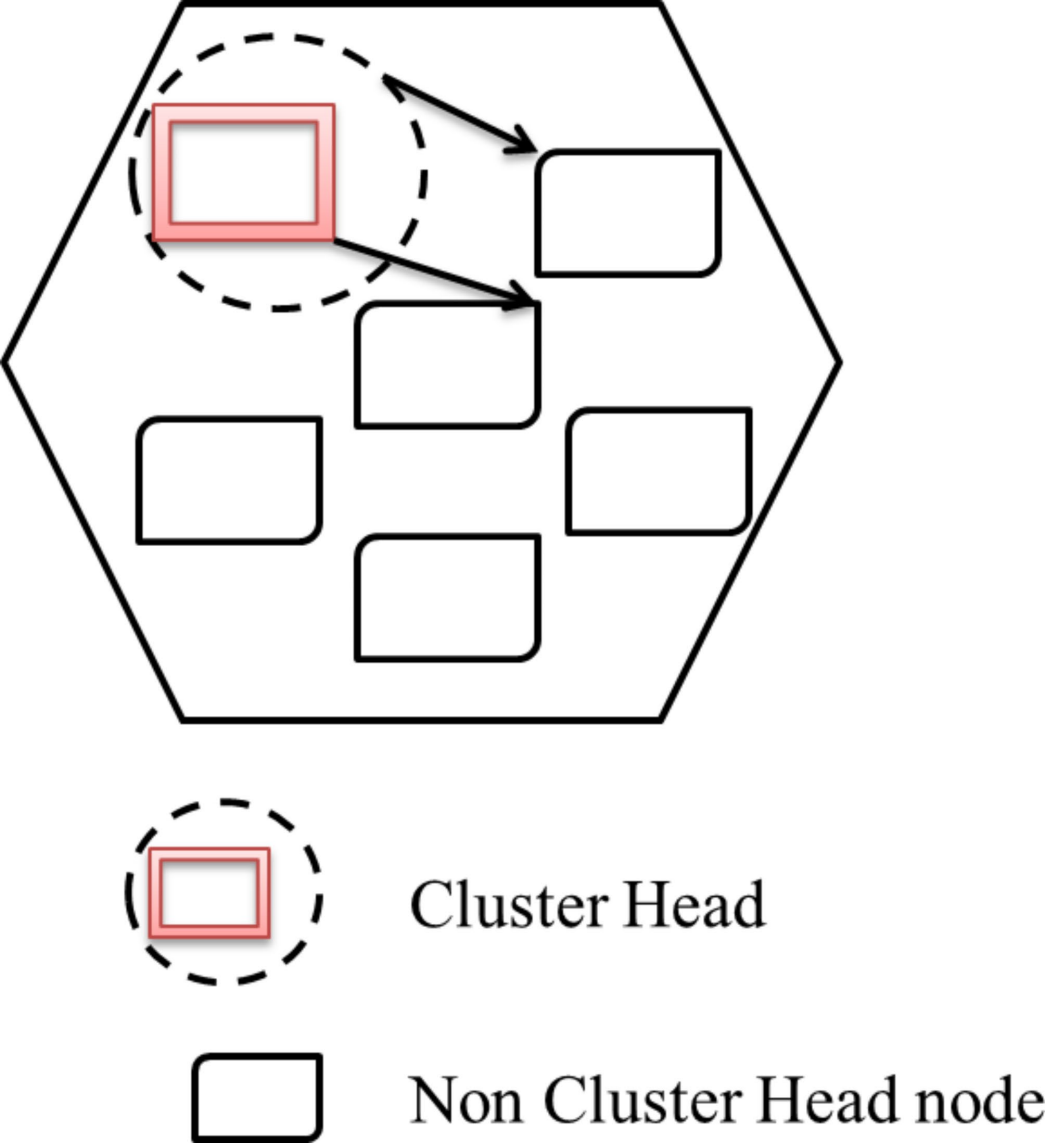
Intra-cluster communication model.

The time period can be operated according to the method of time synchronization. The time synchronization shared the same clock source throughout the entire network channel. This time synchronizing algorithm works the primary node as the system clock for the master-slave concept for the period management. The ethos of time harmonization is that when the beacon frame of a parental node is acknowledged, the substitution node leads the beacon in units of unit number. The fatal node delays the equivalent time period’s integer time, conferring the reasonable discourse’s magnitude. The sample shown in Fig. 15 consists of a 200 ms time period based on the network structure. Figure 16 illustrates the transmission of the beacon frame for a particular time period for all nodes in the network.

### Clustering

The corporal system is converted into a simulated network of interrelated node collections with a cluster-based regulator. There can be one or more regulators per group, and their roles are to create regulator conclusions for group associates and build and dispense depictions of group conditions for outside practice. The etiquette divides the ad hoc network nodes into an amount of overlying or disconnected bunches in a dispersed method. A cluster head (CH) is chosen for each group to sustain cluster association statistics. Inter-cluster directions are exposed vigorously, executing the cluster affiliation information reserved at each cluster head. By gathering nodes into groups, the protocol proficiently diminishes the swamping traffic throughout the direction-finding and speeds up this method. Besides, the etiquette deliberates the presence of one-directional associations and routines these links for both intra-cluster and inter-cluster routing.

Several protocols have been proposed for processing the clustered network^3^. Selecting a good cluster design is solely based on the selection of cluster head selection^47^. Generally, the LEACH protocol is used to determine the cluster head as described in Fig. 16. But it will consume more energy. Therefore, to assuage the excess energy consumption by using the Leach algorithm through the distribution scheme of cluster head assortment outlines. The distribution method has been done using synchronization of each node amid an additional in deciding on an appropriate cluster head. In the case of traffic conditions, the intracluster network uses a dynamic duty cycle. Because Time Division Multiple Access (TDMA) is not an apt method for transmission, it creates a lot of latency. In the context of TDMA, each node will be allocated a time period slot; even if there is no data to convey, it will create to delay. Thereby, it consumes additional power dissipation. Hence, CSMA/CA method is most preferred.

#### Cluster establishment

Subsequently, the selection of cluster head and nodes, the nodes’ transmission will start. Respectively, each node in a cluster will operate according to the allotted time slot, following the three-mode handshake technique. First, if the node wishes to transmit, it will send Ready-to-send (RTS) packet to the CH. Then, the cluster head will obtain information about the node based on the cross-layer technique and decide on the schedule table with the Clear-To-Send (CTS) signifying it to transmit the data acknowledged by the CH over by an ACK^47^.

Energy draining by a collected group associates throughout every single information communication ($\:{E}_{CM})$ could be stated by:

1 $$\:{E}_{CM}={E}_{R}*{T}_{C}+\left(N-1\right)*\left({E}_{I}*{T}_{C}\right)+{E}_{R}*{T}_{CH}+\left({E}_{T}*{T}_{D}\right)$$

Each cluster head obtains N information and regulator sachets. Consequently, the energy disbursed by the cluster head ($\:{E}_{CH})$ is provided by,

2 $$\:{E}_{CH}=\left({E}_{R}*{T}_{CP}+{E}_{R}*{T}_{D}\right)+N({E}_{I}*{T}_{C}+{E}_{T}*{T}_{CH})$$

Table 2 shows the abbreviations of the energy utilisation parameters used in this research.

**Table 2:** Energy utilisation parameters.

Over-all Energy Utilization
N – Number of cluster members within a cluster
$\:{\varvec{E}}_{\varvec{T}}$- Energy Dissipation at the time of broadcast mode
$\:{\varvec{E}}_{\varvec{R}}$- Energy Dissipation throughout the reception mode
$\:{\varvec{E}}_{\varvec{I}}$- Energy Dissipation throughout the indolent mode
C – Time needed for sending/getting a control packet
$\:{\varvec{T}}_{\varvec{D}\:}$– Time needed for sending/getting a data packet
$\:{\varvec{T}}_{\varvec{C}\varvec{H}\:}$– Time reserved by cluster head to transmit a regulator packet.
$\:{\varvec{T}}_{\varvec{C}\:}$– Time needed for transmitting/receiving a regulator packet

## Simulation results

**Fig. 17:**
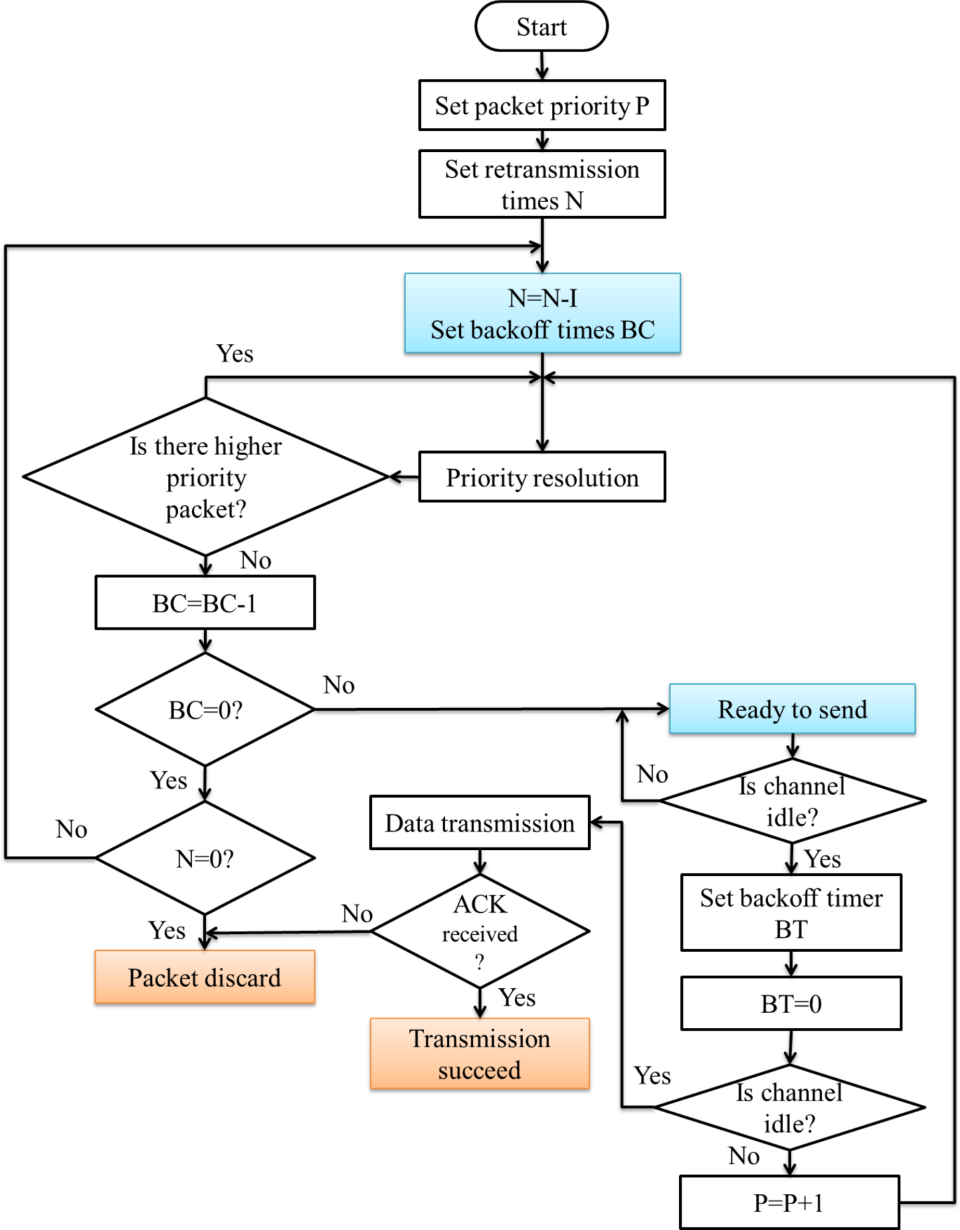
Design flow of proposed intra cluster MAC protocol.

The flowchart of the proposed Media Access Control algorithm has revealed in Fig. 17. The proposed protocol can be simulated and implemented in Matlab software.

**Fig. 18:**
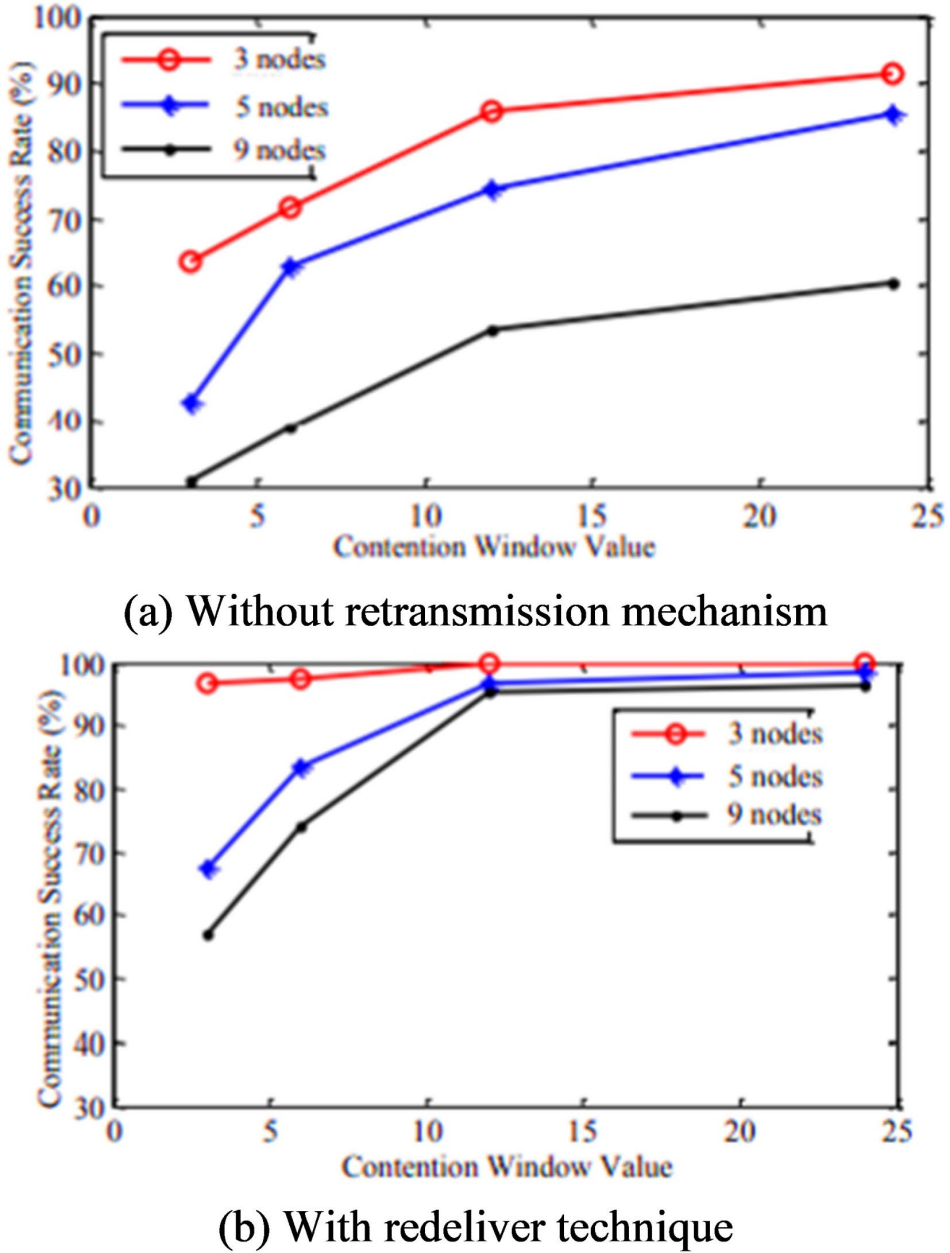
Relationship between communication success rate and contention window value.

**Fig. 19:**
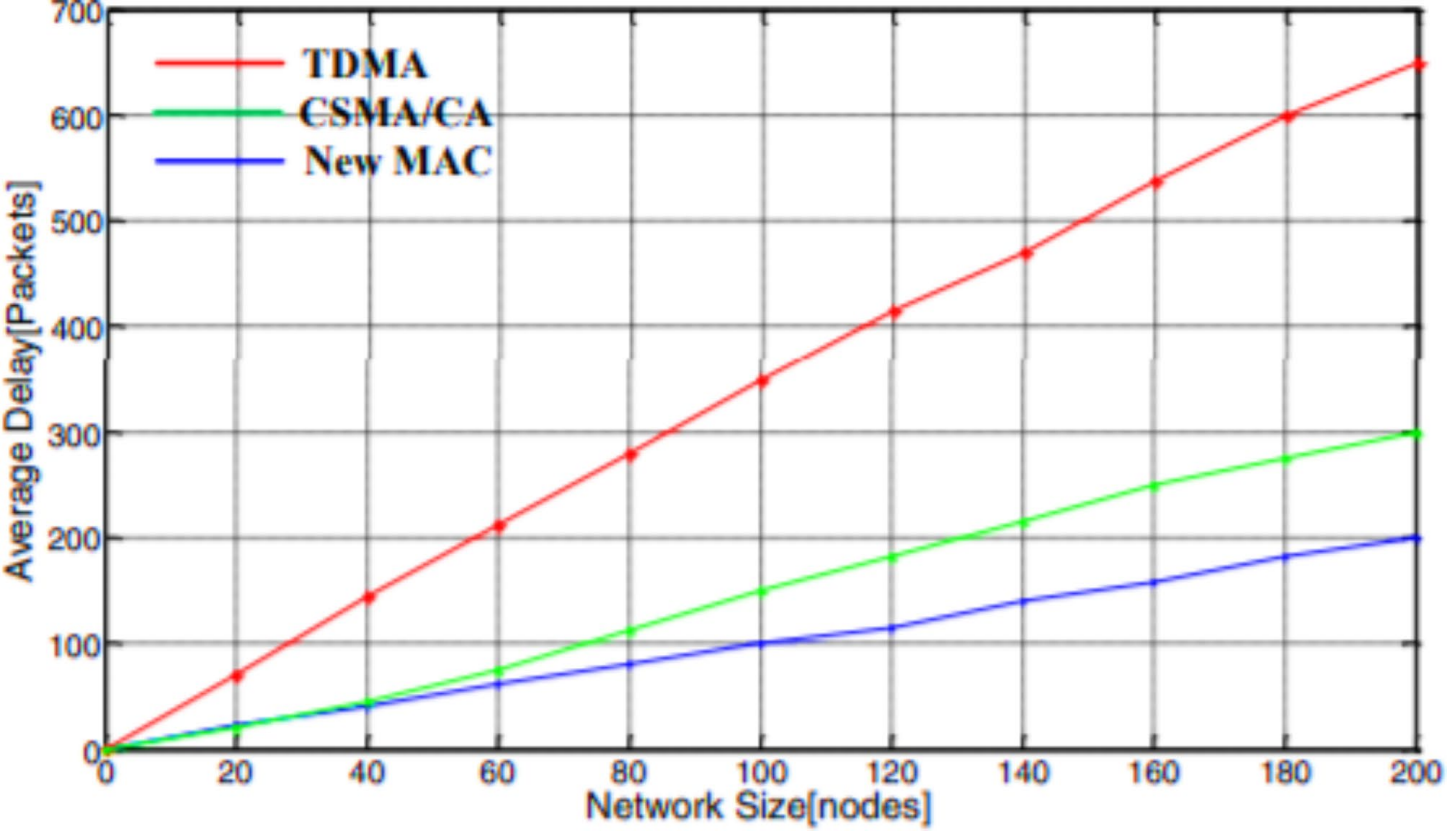
Average transmission delay under different network size.

In a network of different quantities of nodes, all the nodes will transmit 1000 information sachets to the identical nodes with or without redeliver technique built on various arbitrary backoff openings. The average success rate of communication has been determined based on the counts of the successfully received packets. Figure 18 shows the result of the sample test. From the test result, the success rate of the communication has been enhanced with the intensification of the random backoff window, irrespective of the system category. Moreover, Fig. 19 inferred that, with the same amount of arbitrary backoff window, the higher number of nodes in the system would provide degradation in the transmission accomplishment proportion. Also, lower than the similar disorder, the transmission with redeliver protocol improves the results than without the retransmission protocol. In the redeliver technique, when the worth of n is greater than or identical to 12, the transmission accomplishment proportion inclines to 100%, which can encounter the transmission requirements.

The proposed intra-cluster MAC protocol is verified with the parameter of network transmission efficiency. The average delay of the network can be calculated as the time taken to transmit the data successfully to the length of the data packet duration. The average delay of the network will be investigated with the parameter called transmission efficiency. The average transmission delay of the proposed algorithm is calculated for maximum network utilization under different network sizes. The simulation result is taken for 10 different network sizes and set the communication rate of 20 kb/s for the length of a packet is 25 bytes. The results are taken from the simulation for 10,000 successfully transmitted data packets. The data transmission delay is recorded when the network transmission efficiency reaches the maximum range. The simulation results show that the modified intra-cluster MAC protocol provides minor average transmission delay and higher efficiency as the network size expands.

**Fig. 20:**
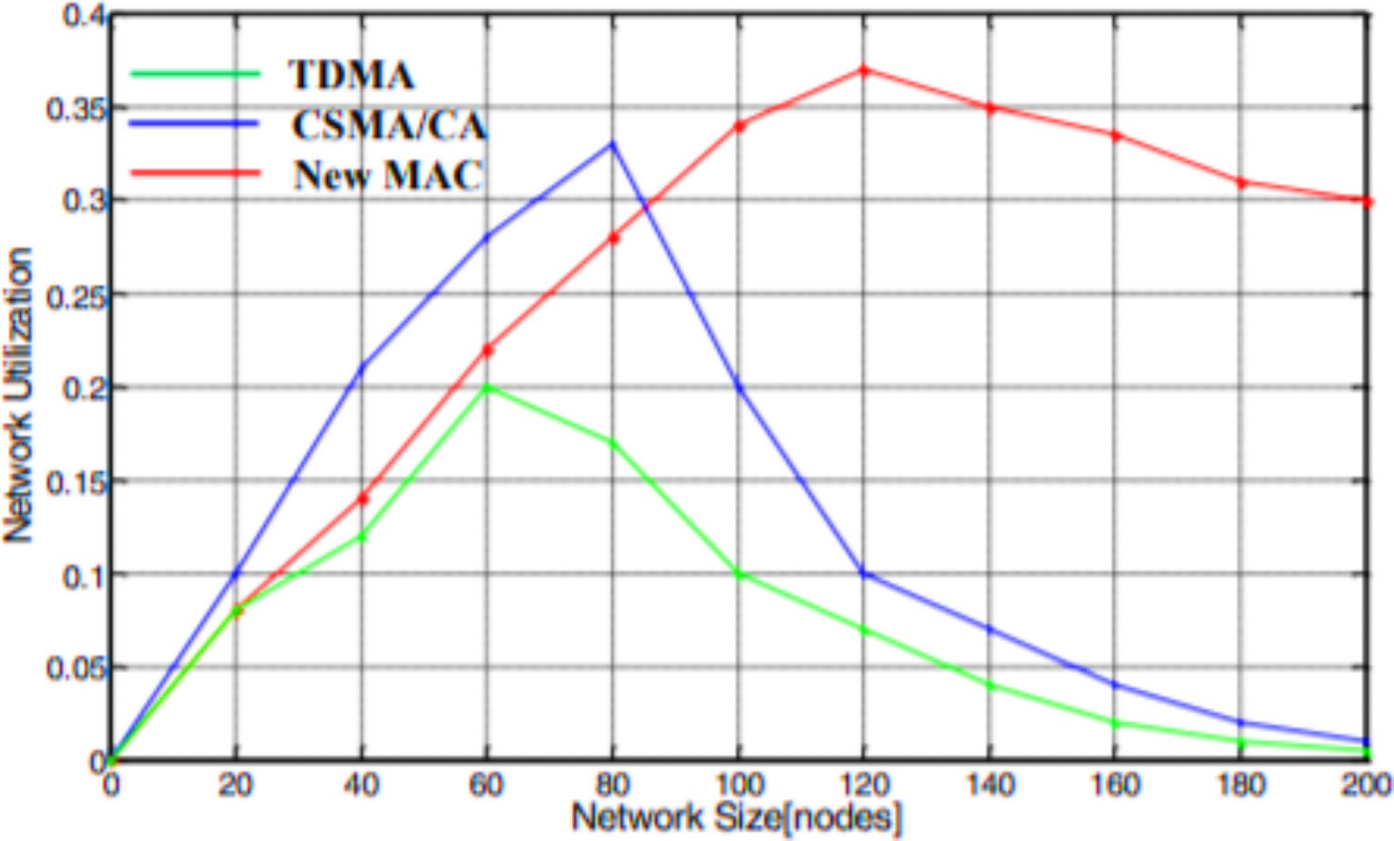
Network usage under dissimilar network size.

Finally, we investigated the network stability parameter, which represents the correlation between network utilization and network size. The obtained results indicate the network utilization report for three protocols, respectively, as shown in Figs. 19 and 20. The simulation results infer that the network resources utilized are weaker for the proposed MAC protocol than the other two protocols due to the protocol overheads. However, as the network size continues to expand, the network utilization of TDMA and CSMA/CA drops sharply, compared to the slight decrease of the new MAC protocol.

## Conclusion

This paper proposed a Modified Intra-cluster MAC algorithm based on time allocation to enhance the reliability of the access nodes in the PLC system. In this procedure, the time period distribution methodology can diminish the possibility of data packet interference. Furthermore, the significance determination technique can assure the appropriateness of information communication. The relentless backoff window can advance the justice of conduit retrieving for the node which flops to entree the former time period. Finally, the recognition technique pledges the information sachet communication appropriately. The software recreation outcomes illustrate that the novel medium access control algorithm projected in this technical write-up can significantly improve the system firmness and data communication competence for big-gauge PLC networks, which is apt for PLC systems.

## Data Availability

The data that support the findings of this study are available from the corresponding author upon reasonable request.
